# Case report: Dirofilarial infection of the face

**DOI:** 10.1016/j.idcr.2024.e02142

**Published:** 2025-01-09

**Authors:** Anne Schneider, Jannik Fasse, Dennis Tappe, Christoph Lübbert, Henning Trawinski

**Affiliations:** a Division of Infectious Diseases and Tropical Medicine, Department of Medicine I, Leipzig University Medical Center, Leipzig, Germany; b Interdisciplinary Center for Infectious Diseases (ZINF), Leipzig University Medical Center, Leipzig, Germany; c Department of Infectious Diseases and Tropical Medicine, Hospital St. Georg, Leipzig, Germany; d Bernhard-Nocht-Institute for Tropical Medicine, Hamburg 20359, Germany

**Keywords:** Subcutaneous filariasis, *Dirofilaria repens*, Microfilaremia, Ukraine

## Abstract

An 18-year-old male patient from Ukraine, living in Germany for 2 years, presented with a painless subcutaneous swelling on the left cheek that had been present for several months. Finally, the diagnosis of subcutaneous dirofilariasis caused by *Dirofilaria repens* was confirmed by 12S rRNA gene PCR and sequencing from tissue by nematode-specific PCRs followed by sequencing after surgical resection of the lesion. Microfilaremia was ruled out and no further treatment was required. Subcutaneous filariasis continues to spread in Central Europe due to climate change, the expansion of vector mosquitoes and the mobility of humans and dogs.

## Highlights

• Diagnosis of subcutaneous Dirofilaria repens infection in a European migrant patient.
• PCR and sequencing confirmed D. repens infection following surgical removal of a cheek lesion.
• Case underlines dirofilariasis spread in Europe due to migration and climate change.
• Microfilaremia was ruled out, and no further treatment was required.

## Case report

**Fig. 1:**
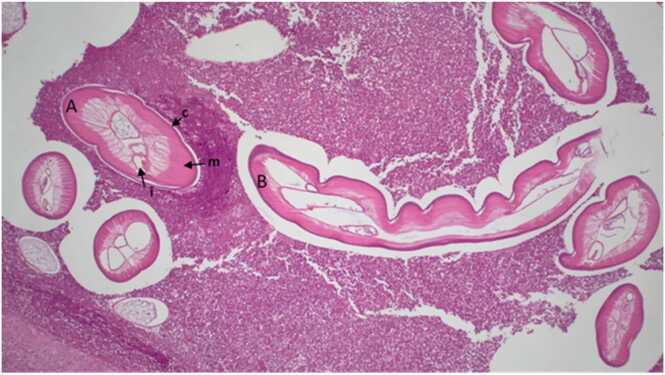
Histopathological sections of a *Dirofilaria repens* helminth surrounded by an eosinophilic inflammation in a subcutaneous lesion of the cheek (courtesy of Prof. Gerhard Taubert, Institute of Pathology at Elsapark, Leipzig, Germany). **A:** cross-section of *D. repens*. **B:** Longitudinal section of *D. repens*. Characteristic morphologic features of *D. repens*: Thick cuticle with longitudinal striations (**c**), musculature (**m**), intestine (**i**).

An 18-year-old male patient was referred to our outpatient clinic with a suspected localized subcutaneous filarial infection. The patient reported persistent swelling of the left cheek and recurrent bleeding after accidentally biting the inside of his cheek five months earlier. An enlarging, painless subcutaneous mass was surgically resected six weeks prior to his presentation at our clinic. Histologic examination revealed numerous sections of a nematode larva surrounded by eosinophilic inflammation (Fig. 1). Onchocerciasis was suspected by primary care providers and the patient was given a single dose of ivermectin 200 µg/kg body weight and doxycycline 200 mg per day for seven days.

We suspected a different form of subcutaneous filariasis and requested the removed tissue to be sent to the German National Reference Center for Tropical Pathogens at the Bernhard Nocht Institute, Hamburg, where the nematode larva was identified as *Dirofilaria (D.) repens* by positive nematode-specific 12S rDNA [1] and cytochrome oxidase [2] PCRs followed by sequencing (98 % and 100 % homology to sequences in GenBank from Greece (MK192091) and Hungary (KX265053), and from Croatia (KX265049) and Italy (KX265048), respectively; BLAST analysis (https://blast.ncbi.nlm.nih.gov/Blast.cgi)).

At the time of presentation, the patient denied any signs of systemic infection such as fever, rash, night sweats or lymph node swelling. Clinical examination revealed no pathologic findings, and there was no skin or soft tissue swelling. Blood tests showed no eosinophilia or elevated inflammation markers. A serologic in-house screening ELISA for filarioid infections using crude *Dirofilaria immitis* antigen was positive (51 arbitrary units, normal < 10; Bernhard Nocht Institute, Hamburg). The assay has a diagnostic sensitivity of 85 % and a specificity of 82 % for any filarioid infection as determined by using 28 well-defined sera from patient cohorts with microscopically or molecular confirmed filariasis (incl. 5 dirofilariasis cases) versus patients with other nematode infections, other diseases, or healthy subjects. Microfilaremia was ruled out by microscopic examination of approximately 22 mL of blood plasma taken around noon; further therapy was therefore not considered necessary.

The patient originates from northern Ukraine and had been living in Germany (near Leipzig, Saxony) for two years. He left Ukraine about 1.5 years before he noticed the swelling on his cheek. During his travel from Ukraine to Germany, he stayed only a few hours in Poland and spent one night in a friend’s apartment with two dogs and several cats in Dresden, Saxony. He had never owned a pet dog but reported that after the outbreak of war in his home country, he spent five days in a bunker with about 100 other people and a few pets. He denied further stays abroad, particularly in tropical or subtropical countries.

## Discussion

Dirofilariasis is a zoonotic disease transmitted by mosquitoes and caused by filarioid helminths of the genus *Dirofilaria*. The most important species in terms of incidence and geographical distribution are *D. immitis* and *D. repens*, which cause pulmonary and subcutaneous dirofilariasis, respectively. Dogs and other canids are the main hosts and reservoirs of *D. immitis* and *D. repens*. Although they can also infect a variety of vertebrates, including cats and other felids. Humans can become accidental hosts. Mosquito species from different genera (*Anopheles, Aedes, Culex, Coquillettidia*) act as vectors and intermediate hosts by ingesting circulating microfilariae while feeding on infected hosts [3]. Larval development in the mosquito is sensitive to environmental factors such as temperature.

*Dirofilaria immitis* has a worldwide distribution. In final hosts, the adult worms live inside pulmonary arteries and the right ventricle, causing chronic inflammation with severe cardiorespiratory symptoms (canine heartworm disease). In humans, the infection typically presents as an incidental finding of a solitary, well-defined pulmonary nodule ("coin lesions") in asymptomatic patients. Some patients report preceding symptoms of pneumonitis, such as cough [4]. There are no reports of microfilaremia in human infections with *D. immitis* [5]. Only about 25 cases have been reported in Europe in recent decades [6].

*Dirofilaria repens* is the most common species causing human dirofilariasis and has been detected on all continents except the Americas [6], [7], [8]. It has been considered endemic in southern Europe since the early 20th century and has recently been found in many central and eastern European countries as well as in the Balkans [6]. Due to climate change, the spread of competent vector mosquitoes like *Aedes albopictus*, the movement and trade of companion dogs throughout Europe and increased contact between humans and pet animals, the number of autochthonous human cases in Western and Eastern Europe is currently on the rise [9], [10], [11], [12]. From 1977 to 2016, more than 3500 infections were reported in Europe [6]. In Ukraine, where reporting of dirofilariasis cases has been mandatory since 1975, a total of 1533 human cases were registered from 1975 to 2012, with 1465 cases reported since 1997 and the number rising steadily during this period [13].

The adult stages of *D. repens* are usually found in the subcutaneous and intermuscular connective tissue and have a lifespan of up to ten years [3]. Infections in dogs usually remain asymptomatic and occasionally manifest as subcutaneous, non-painful nodules, erythema, pruritus, alopecia or papules [5]. In humans, the parasites usually do not reach the sexually mature adult stage, and in most cases only an immature, pre-adult worm forms a subcutaneous nodule weeks to months after infection, often located in the head and neck region or in the subconjunctival tissue [6]. While subcutaneous and ocular manifestations are the most common presentations of *Dirofilaria repens* infections in humans, systemic manifestations, though rare, have been documented [7], [8]. Cases of eosinophilic meningoencephalitis associated with *D. repens* have been reported, highlighting its potential to cause central nervous system involvement [14]. This condition likely results from the aberrant migration of larvae. Additionally, cases of pulmonary or pleural dirofilariasis have been noted, where nodules resembling malignancies were observed on imaging studies [7], [15]. These presentations underscore the need for heightened clinical awareness in endemic and non-endemic regions. In rare cases, mature females with microfilariae have been detected, and microfilaremia has been confirmed by microscopy or PCR from blood plasma, although detection of both sexes in the same patient has not yet been demonstrated [3], [8], [9]. A blood test for microfilariae is strongly recommended for human infections with *D. repens* infections [6]. Usually the infestation can be resolved by surgical removal of the subcutaneous nodules or the parasite from the conjunctiva and no further medical treatment is needed [3]. In cases where microfilaremia is detected, additional treatment with anthelmintics (albendazole, ivermectin) or doxycycline is recommended [8].

With this case, we aim to highlight dirofilariasis as an important differential diagnosis in patients presenting with localized subcutaneous or subconjunctival swelling and inflammation, especially in the context of migration from highly endemic regions such as Ukraine to Central Europe and the possible spread of dirofilariasis in Europe due to climate change. Although rare, microfilaremia should always be ruled out and treated when detected.

## CRediT authorship contribution statement

**Jannik Fasse:** Writing – review & editing. **Anne Schneider:** Writing – original draft, Project administration, Conceptualization. **Henning Trawinski:** Writing – review & editing, Supervision, Conceptualization. **Christoph Lübbert:** Writing – review & editing. **Dennis Tappe:** Writing – review & editing.

## Author agreement

I hereby confirm that all authors have reviewed and approved the final version of the manuscript titled “Case report: Dirofilarial infection of the face”, being submitted to *ID Cases* for consideration.

## Ethical approval

Not required.

## Consent

The patient has consented to the submission of the case report to the journal.

## Funding Source

We acknowledge support from 10.13039/501100008678Leipzig University for Open Access Publishing.

## Conflict of Interest

None.

## Declaration of Competing Interest

The authors declare that they have no known competing financial interests or personal relationships that could have appeared to influence the work reported in this paper.
